# Size-Dependent Superconducting Properties of In Nanowire Arrays

**DOI:** 10.3390/nano12224095

**Published:** 2022-11-21

**Authors:** Alexey A. Noyan, Yevgeniy A. Ovchenkov, Valery V. Ryazanov, Igor A. Golovchanskiy, Vasily S. Stolyarov, Eduard E. Levin, Kirill S. Napolskii

**Affiliations:** 1 Moscow Institute of Physics and Technology, 141700 Dolgoprudny, Russia; 2 Lomonosov Moscow State University, 119991 Moscow, Russia; 3 Institute of Solid State Physics RAS, 142432 Chernogolovka, Russia; 4 National University of Science and Technology MISIS, 119049 Moscow, Russia

**Keywords:** superconducting nanowires, anodic aluminum oxide, indium, electrodeposition, magnetization curve

## Abstract

Arrays of superconducting nanowires may be useful as elements of novel nanoelectronic devices. The superconducting properties of nanowires differ significantly from the properties of bulk structures. For instance, different vortex configurations of the magnetic field have previously been predicted for nanowires with different diameters. In the present study, arrays of parallel superconducting In nanowires with the diameters of 45 nm, 200 nm, and 550 nm—the same order of magnitude as coherence length *ξ*—were fabricated by templated electrodeposition. Values of magnetic moment *M* of the samples were measured as a function of magnetic field *H* and temperature *T* in axial and transverse fields. *M*(*H*) curves for the arrays of nanowires with 45 nm and 200 nm diameters are reversible, whereas magnetization curves for the array of nanowires with 550 nm diameter have several feature points and show a significant difference between increasing and decreasing field branches. Critical fields increase with a decrease in diameter, and the thinnest nanowires exceed bulk critical fields by 20 times. The qualitative change indicates that magnetic field configurations are different in the nanowires with different diameters. Variation of *M*(*H*) slope in small fields, heat capacity, and the magnetic field penetration depth with the temperature were measured. Superconductivity in In nanowires is proven to exist above the bulk critical temperature.

## 1. Introduction

The development of superconducting nanoelectronic devices has remained an important task of studies in recent years. Key elements of the devices are Josephson junctions with tailored properties [1] and elements based on superconducting weak links, such as SQUID [2,3] and quantum interference effect transistors [4]. There is a constant endeavor to improve the properties of superconducting devices: the increase in breakdown current [5] and stability in the air [6], the rise in working temperature [7], and the increase in magnetic field sensibility [8]. Improvement potential is limited for nanostructures fabricated by sputtering, as this technology has significant limitations in controlling the crystallinity of the samples. For instance, magnetron sputtering commonly produces fine crystalline films with a grain size of less than several nanometers. Morphology of the sputtered films may change significantly later, for example, in the case of thermal treatment, but additional difficulties with the stability of chemical composition and morphology of all elements in the device at high temperatures arise. Therefore, the use of coarse-crystalline superconducting nanowires obtained by templated electrodeposition [7,8,9,10,11,12] as the weak link is a possible step to improve superconducting devices. The templated electrodeposition has been successfully used for many metals and is being actively developed [13,14,15,16,17,18]. If deposition parameters are adjusted properly, the fabrication of single-crystalline nanowires is possible. Thereby Josephson junctions based on electrodeposited nanowires have superior characteristics compared to junctions fabricated by sputtering [7]. Other advantages of electrodeposition are a wide range of deposition solutions and the ability to obtain multi-segmented nanowires [19].

Studies of superconductivity In nanowires are essential for assembling nanoelectronic devices. Nanowires have shown several unique features of electron transport; one of the questions is the interplay of superconductivity and ferromagnetism In nanowires. The works [12,20] showed a decrease in resistance in 1-μm-long Co nanowires used as a weak link. That is a great length compared to a scale of 1 nm magnitude order at which proximity effect is observed in bulk ferromagnets. In [21], it was shown that Pb nanowire with the admixture of Co exhibits both superconducting and ferromagnetic properties. Another peculiar feature is the superconductivity of single-crystalline Bi nanowires at temperatures up to 1.3 K [16], whereas the critical temperature of the bulk Bi is as low as 0.53 mK [22].

**Figure 1:**
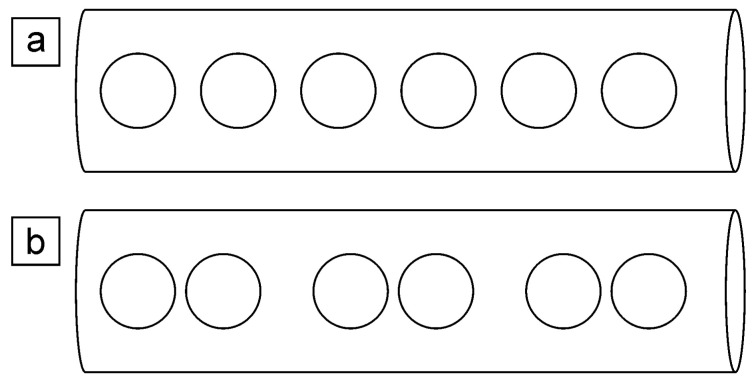
Scheme of the simplest possible vortex configurations In nanowire according to the simulation [24]: 1D lattice of single-quantum vortices (**a**) and a row of single-quantum vortices divided into pairs (**b**). Circles are superconducting vortices.

Magnetic field distribution in superconducting nanowires is another open question. Three states of superconductor are distinguished: Meissner state, where the magnetic field is completely expelled, vortex state with a lattice of single quantum vortices, and intermediate mixed state in which more sophisticated field configurations are possible [23]. The picture is complicated in the case of nanowires because of geometrical limitations. In [24], a numerical simulation was conducted for nanowires of a type I superconductor in the transverse magnetic field. Diameters of the nanowires from 5 $\xi_{0_{b}}$ to 50 $\xi_{0_{b}}$ were studied, where $\xi_{0_{b}}$ is bulk coherence length. The simulation showed that nanowires could demonstrate type-II magnetic response. Different distributions of the penetrated field In nanowires depending on the diameter are predicted; the configurations differ significantly from ones observed in bulk superconductors and thin films [25]. Modeled dependence of magnetization *M* as a function of magnetic field strength *H* is nonmonotonic with several features related to shifts between different configurations. Two vortex configurations of the penetrated magnetic field were observed in simulation for the thinnest nanowires analyzed (Figure 1): 1D lattice of single-quantum vortices (a) and a row of single-quantum vortices divided into pairs (b). It is worth noting that the theoretical results [24] have not been proved experimentally yet.

Magnetization behavior of β-Ga nanowire array with a diameter of 140 nm ($2.3\;\xi_{0_{b}}$) in the transverse field was studied experimentally [26]. *M*(*H*) curves demonstrated hysteresis and type-II-like behavior. The authors interpreted the observed *M*(*H*) dependences as the sign of vortex configuration illustrated in Figure 1a. Magnetization of a single Pb nanowire with 390 nm ($4.5\xi_{0_{b}}$) diameter was measured locally by the Hall probe [27]. *M*(*H*) curve had hysteresis at low temperatures, which disappeared at temperatures closer to the *T_C_*. The simulation was made for low temperatures, which showed the configuration of the penetrated field as a lattice of single-quantum vortices similar to the configuration in Figure 1a. Simulated data agreed with the experimental results. Magnetization *M*(*H*) for Pb nanowires with 200 nm ($2.3\;\xi_{0_{b}}$) diameter were measured in [28]. The studies [27,28] showed the enhancement of critical magnetic fields In nanowires.

In the present work, arrays of In nanowires were analyzed. Specific properties of In are large coherence length and the presence of a thin (about 4 nm) surface oxide layer preventing the metal core from further oxidation in the air [29]. These properties make In promising for applications in superconducting electronics. Bulk In is a type I superconductor with a critical temperature *T_C_* = 3.408 K and a zero-temperature critical field *H_C_*(0) = 281.5 Oe [30]. The mean value of coherence length measured in bulk indium is $\xi_{0_{b}}$ = 264 nm, and the mean penetration depth is $\lambda_{0_{b}}$ = 53 nm [31,32,33,34,35,36,37]; all values of $\lambda_{0_{b}}$ and $\xi_{0_{b}}$, which were averaged, are listed in Appendix A. Experimentally observed temperature dependence of critical field *H_C_* is in good agreement with the general law [30]:

(1) $$H_{C}\left(T\right)=H_{C}\left(0\right)\left(1-{\left(\frac{T}{T_{C}}\right)}^{2}\right)\;$$

Different methods to fabricate In nanowires were proposed in studies [38,39,40,41,42,43,44,45,46,47,48,49], including vacuum sputtering in V-shaped silica grooves [40,41], oblique angle deposition [42], and injection of molten metal into a porous template under pressure [48]. Templated electrodeposition of In nanowires [38,39,49] is the most promising method as it allows one to set geometrical parameters of the nanowires precisely and, at the same time, to fabricate single-crystalline nanowires after adjustment of deposition conditions. To the best of our knowledge, superconductivity in In nanowires has not been studied yet. However, several studies on In nanoparticles have been conducted [50,51], where molten indium was injected into the porous glass under pressure. The characteristic size of the particles was determined as a function of the pressure used, but the shape of the particles and size distribution were not defined. The measurements of magnetization curves showed a significant increase in critical fields with a decrease in indium particle size. Additionally, the rise of critical temperature was observed compared to the bulk In for the particles with a characteristic size of 3.1 nm: the critical temperature was 0.75 K higher than the bulk critical temperature. For particles with a size of 25 nm, the increase in critical temperature within experimental error was measured.

## 2. Materials and Methods

Arrays of superconducting In nanowires were fabricated by electrodeposition technique using polycarbonate track-etched membranes (Whatman, Little Chalfont, UK; pore diameter of 550 nm, thickness of 20 microns) and anodic aluminium oxide (AAO) as templates. Porous AAO films were prepared by anodization of a high-purity aluminium foil (99.99%) with a thickness of 100 μm. Prior to anodization, the foil was electrochemically polished to a mirror finish in a solution containing 13 M H_3_PO_4_ and 1.85 M CrO_3_ at 80 °C as described elsewhere [52]. The aluminium electrode was polarized 40 times for 3 s at an anodic current density of 0.5 A cm^−2^ with an interpulse interval of 40 s. Two series of AAO templates with pore diameters of 45 nm and 200 nm were fabricated in different anodizing regimes. The film with a pore diameter of 45 nm was formed using two-step anodization procedure in 0.3 M oxalic acid (Chimmed, Moscow, Russia) at a voltage of 40 V and an electrolyte temperature of 0 °C. During the first anodization step, a 10 μm thick sacrificial alumina layer was formed. This layer was then selectively dissolved in an aqueous solution containing 0.5 M H_3_PO_4_ and 0.2 M CrO_3_ at 70 °C for 30 min. The second anodization under the same conditions as the first one was stopped when AAO thickness reached 40 μm. The AAO films with a pore diameter of 200 nm and a thickness of 46 μm were formed in 0.1 M phosphoric acid (Chimmed, Moscow, Russia) at a voltage of 195 V and an electrolyte temperature of 0 °C. After anodizing, the porous oxide films were washed repeatedly in deionized water and dried in air. The residual Al was dissolved in a solution of Br_2_ in CH_3_OH (1:10 volume). The barrier oxide layer was removed by chemical etching in 3 M H_3_PO_4_ solution at room temperature with the electrochemical detection of the pore opening moment [53].

**Figure 2:**
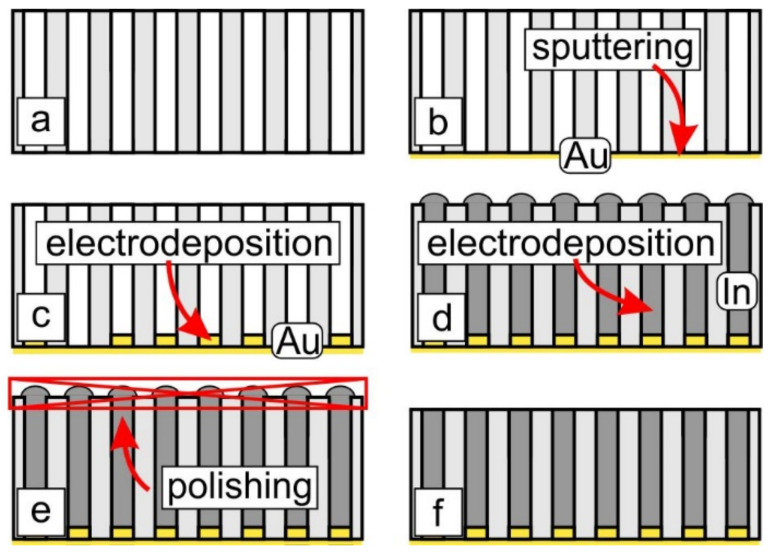
A flowchart of In nanowire array preparation process. (**a**) The original porous template. (**b**) Sputtering of Au layer on the porous template. (**c**) Templated electrodeposition of short Au nanorods. (**d**) Templated electrodeposition of In until complete filling of the pores by the metal. (**e**) Mechanical polishing of the top part of the nanocomposite to remove In from sample surface. (**f**) Resulting nanocomposite.

Electrodeposition of In was performed in a three-electrode cell in potentiostatic mode using a PGSTAT100N (Metrohm Autolab, Utrecht, The Netherlands) instrument. AAO and track-etched membranes were coated with a 300-nm-thick Au layer using a magnetron sputtering system. This continuous gold layer served as a current collector. Before In electrodeposition 2 μm long Au nanorods were deposited to prevent the formation of superconducting material under a porous template. For this purpose, a commercial electrolyte (Ecomet, Moscow, Russia) containing 0.04 M Au[CN]_2_^−^ in citrate buffer (pH = 6) was used. A Pt wire ring with a diameter of ca. 2 cm was used as a counter electrode. The saturated (KCl) Ag/(AgCl) electrode connected with the cell via Luggin capillary served as the reference electrode; deposition potential was −1.0 V. Indium was deposited from a solution containing 0.1 M In(SO_3_NH_2_)_3_ and 1.25 M Na(SO_3_NH_2_) (pH = 2.1). An indium rod served as the reference electrode. An indium ring was used as a counter electrode. A deposition potential of −0.4 V was used. To increase the filling factor during the electrodeposition of In in AAO films the template surface was cleaned repeatedly by electrolyte flow. Indium in track-etched membranes was deposited to half of the membrane thickness. After the electrodeposition, the top side of the AAO films and the current collector side of the track-etched membrane were mechanically polished to remove indium from the surface (Figure 2). This procedure allows one to obtain samples with metal solely in the pores.

Magnetization measurements were carried out using an MPMS XL (Quantum Design, Darmstadt, Germany) SQUID magnetometer. The phase composition of the samples was characterized with a Aeris (Malvern Panalytical, Malvern, UK) tabletop X-ray diffractometer (CuKα radiation, PIXcel3D detector). The morphology of the porous oxide films and track-etched membranes was characterized using a NVision 40 (Carl Zeiss, Oberkochen, Germany) scanning electron microscope. Before SEM analysis, the samples were coated with a 5-nm-thick conductive layer of chromium using a Q150T ES (Quorum Technologies, Lewes, UK) sputter coater. The mean porosity of AAO films with a pore diameter of 45 nm was measured using the birefringence method [54].

## 3. Results and Discussion

**Figure 3:**
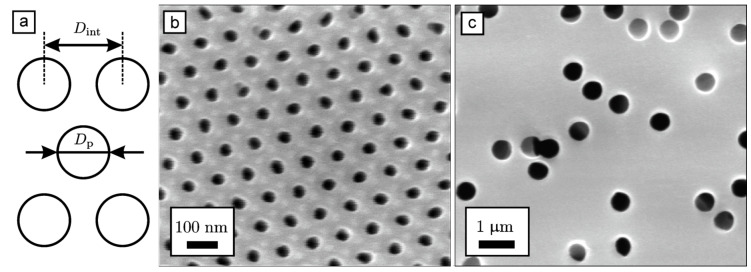
Scheme of pore arrangement (**a**) and SEM images of the surface of AAO film s45 (**b**) and polymer track-etched membrane s550 (**c**).

**Figure 4:**
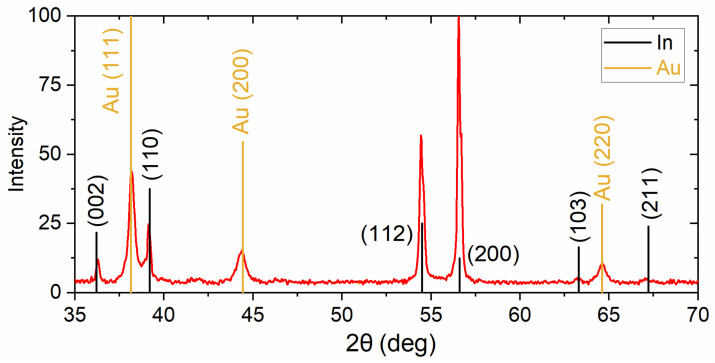
XRD pattern for AAO/In nanocomposite containing array of nanowires with 200 nm diameter.

Three kinds of superconducting In nanowire arrays with various diameters of the nanostructures were prepared. For this purpose, two types of the AAO films and a polymer track-etched membrane with cylindrical pores aligned perpendicular to the surface were used as templates. The morphology of the templates was characterized using SEM (Figure 3). The parameters of the templates calculated from the statistical analysis of the SEM images are listed in Table 1. AAO films possess 2D hexagonal ordered pore arrangement in the plane of the film and narrow interpore distance distribution. Conversely, pores in the polymer track-etched membrane had no positional order; the positions of the pores are random with few pore overlaps; moreover, deviation of pores from the normal to the surface of the track-etched membrane is possible. The porosity of the sample s45 was measured with the optical birefringence method, which is more precise than SEM [54]. Diameters of In nanowires equal to the pore diameter D_p_ and had the same order of magnitude as $\xi_{0_{b}}$ and $\lambda_{0_{b}}$, therefore geometrically limited superconducting states could be studied. Values of pore volume V in samples were calculated from the geometrical parameters (Table 1), taking into account a diameter of the porous electrode of 1.2 cm, identical for s45, s200, and s550. X-ray diffraction showed (Figure 4) that nanowire arrays are featuring preferential orientation along the <100> axis.

**Figure 5:**
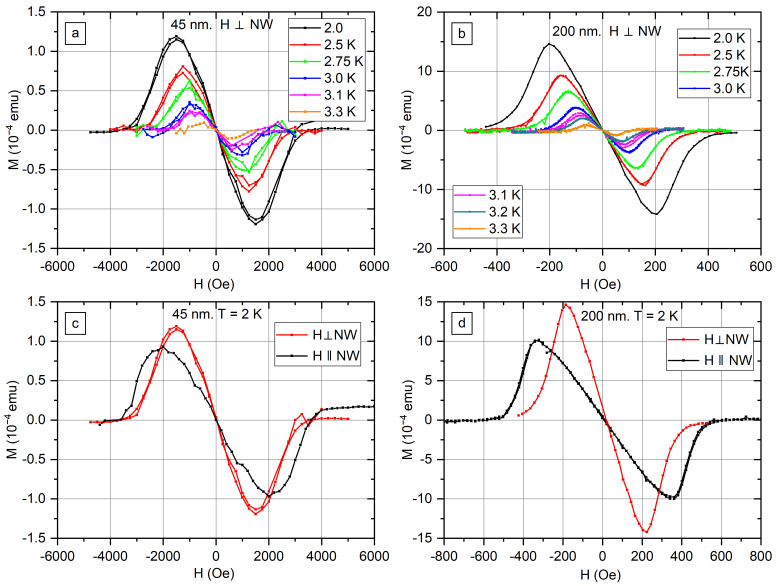
The magnetic moment *M* as a function of transverse magnetic field *H* for In nanowire arrays with nanowire diameters 45 nm (**a**) and 200 nm (**b**). The comparison of *M*(*H*) curves measured at 2 K temperature for the same sample in transvers and axial fields: s45 (**c**) and s200 (**d**).

Figure 5 displays magnetic moment *M* of In nanowire arrays s45 and s200 as a function of magnetic field *H* at temperatures from 2 K to 3.3 K. *M*(*H*) curves measured for the field perpendicular to the long axis of the nanowires are shown in Figure 5a,b. It is worth noting that all curves measured for the nanowires with diameters of 45 nm and 200 nm are reversible, values of magnetic moment *M* measured in increasing and decreasing magnetic fields are the same at each field value. The diameter of 200 nm is almost equal to $\xi_{0_{b}}$ and 45 nm diameter is several times smaller than the coherence length of bulk In. The absence of vortex states in thin nanowires is the probable explanation of reversibility.

Figure 5c,d compares curves measured in transverse and axial fields at 2 K. Curves measured in transverse fields have larger maximum values of M and less sharp decline after the maximum. A different demagnetization factor is one of the reasons for such a difference in the shape of magnetization curves. The demagnetization factor of the cylinder in the axial field is 0, and the demagnetization factor in the transverse field is ½. The macroscopic shape of the sample is another factor that influences the shape of the magnetization curve.

*M*(*H*) curves measured for nanowires parallel to the field *H* at temperatures from 2.0 K to 3.6 K are shown in Figures S1 and S2. Weak diamagnetic response is visible on the *M*(*H*) curve for s200 at *T* = 3.5 K with no response at *T* = 3.6 K. Therefore, superconductivity in In nanowires is possible at temperatures above a bulk critical temperature of In (*T_C_* = 3.408 K).

**Figure 6:**
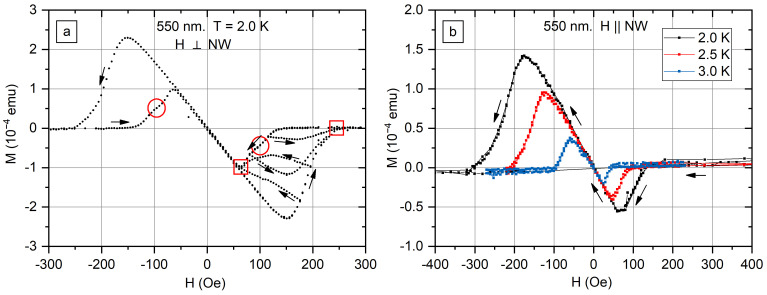
The magnetic moment *M* of 550-nm-diameter In nanowire array as a function of transverse (**a**) and axial (**b**) magnetic field *H*. Arrows show how the field was changing. Two joining points of minor loops are marked with red squares. The feature of the *M*(*H*) curve in the transverse field, which is absent in case of axial field, is marked with the red circle in panel (**a**).

The magnetization curves for arrays of nanowires with 550 nm diameter recorded in transverse and axial fields are shown in Figure 6a and Figure 6b, respectively. For a clear view, only the decreasing field branch is presented in Figure 6b. In contrast to s45 and s200 samples, *M*(*H*) curves for s550 show hysteresis. Higher magnetic moments are reached in the increasing field branch, and *M* remains zero in a larger field interval on the decreasing field branch. However, no hysteresis is observed in small fields. The exit of a magnetic field from the nanowire is suppressed. The probable explanation of the observed behavior is the formation of a vortex state In nanowires with a surface barrier causing the hysteresis. Another explanation may be the pinning of vortices on grain boundaries. It should be noted that *M*(*H*) curves for s550 in the transverse field are qualitatively similar to measured ones for the array of β-Ga in [26].

Figure 6a shows a set of minor loops, directions of field *H* changing are shown with arrows. All the minor loops for increasing fields join with the main loop at the same point, in which *M* = 0. All the minor loops for decreasing fields join the main loop in another point, corresponding to the maximum magnetization on decreasing field branch. Two joining points of minor loops are marked with squares (Figure 6a).

It is worth noting that the feature present in both positive and negative magnetic fields on the *M*(*H*) curve for s550 in the transverse field, where the derivative changes twice (see red circle in Figure 6a). It was observed on curves recorded at temperatures 2.0 K and 2.5 K (Figure S3). The feature corresponds to approximately ½ of the maximum *M* value. No such features are present on the *M*(*H*) curves in the axial field (Figure 6b). A possible explanation of the observed feature is a transition between the two simplest vortex configurations (Figure 1), predicted in the simulations in [24].

**Figure 7:**
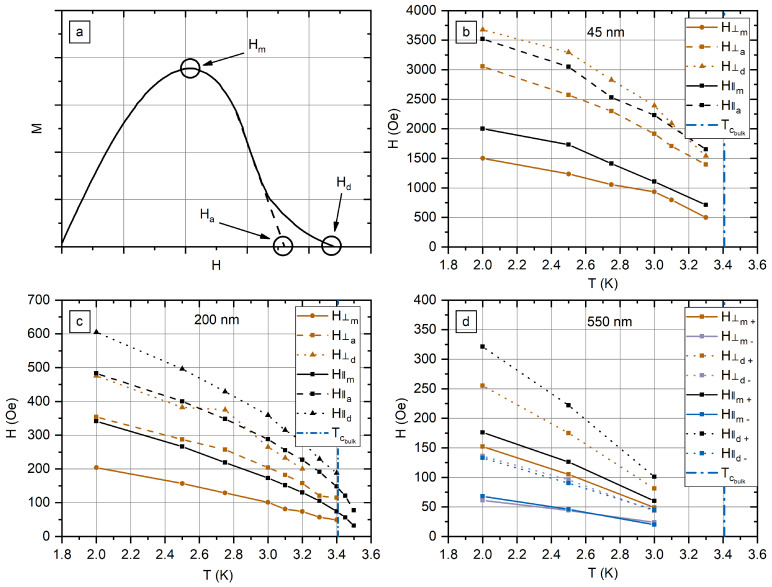
(**a**) Schematic illustration of the critical fields *H_m_*, *H_a_*, and *H_d_* on *M*(*H*) curve. Values of fields measured for the arrays of In nanowires with a diameter of 45 nm (**b**), 200 nm (**c**), and 550 nm (**d**). Values measured for increasing in modulus field *H* have index +, whereas values measured for decreasing in modulus field *H* have index −.

There are three critical fields *H_m_*, *H_a_*, and *H_d_* on the *M*(*H*) curves that were measured as shown in Figure 7a. *H_m_* is the field of the maximum of magnetization, *H_a_* is the extrapolation of the linear region of *M* decline, and *H_d_* is the field at which *M* becomes zero. Figure 7b–d presents characteristic fields for s45, s200, and s550 samples. Values of the critical fields for the array of In nanowires with a diameter of 550 nm were calculated separately for increasing (+) and decreasing (−) branches.

The analysis of the critical fields shows that superconductivity in In nanowires is possible in much higher fields than in bulk indium. *H_m_* in 550 nm nanowire array are close to the bulk critical field of In, $H_{C_{b}}$ (2 K) = 180 Oe (see Appendix A), *H_d_* exceeds bulk critical field. The critical fields increase with the decrease In nanowire diameter. In the transverse field, *H_m_* is 200 Oe in 200 nm nanowires and 1500 Oe in 45 nm nanowires. The superconducting response is detected in the field of more than 3500 Oe, exceeding the bulk critical field by 20 times. The shape of the *M*(*H*) curve with a slow decay in high fields indicates residual surface superconductivity.

**Figure 8:**
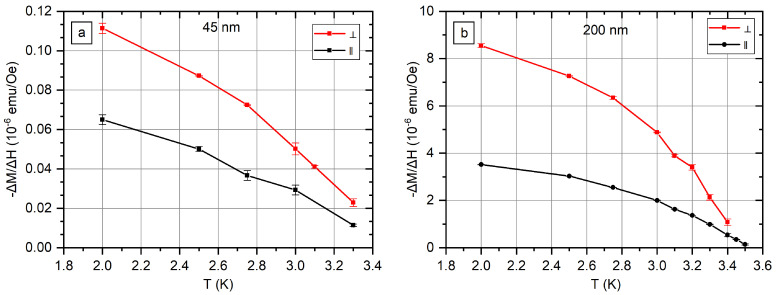
The slope of the linear part of *M*(*H*) curve observed at small fields for s45 (**a**) and s200 (**b**).

*M*(*H*) curves measured for s550 at temperatures 2 K, 2.5 K, and 3.0 K (Figure 5) coincide on the linear region in small fields. The nanowires with a 550 nm diameter are thick enough to perform the Meissner effect with full expulsion of the magnetic field, and the slope is adjusted by the volume of the superconductor. In contrast to the s550 sample, the slope of the linear *M*(*H*) region in the s45 and s200 samples depends on the temperature. Figure 8 shows the slopes calculated for s45 and s200. The metal fraction in the nanocomposites s45 and s200 is close (Table 1); however, the slope differs by two orders of magnitude (Figure 8). The reason is that *λ* is of the same order of magnitude as the nanowire diameter, and full expulsion of the magnetic field does not take place. Therefore, in s45, the sample magnetic field penetrates almost the full nanowire diameter. The small field region is linear, as the configuration of the magnetic field does not change with rise of the field. *λ* increases with the rise of temperature, accordingly the slope decreases with temperature.

For nanowires with a diameter smaller than *λ* in the case of an axial field, the magnetic moment is derived with the equation:

(2) $$\frac{M}{V}=\frac{R^{2}H}{32\pi \lambda^{2}}$$

Magnetic slopes at different temperatures are connected with the equation:

(3) $${\left(\frac{\Delta M}{\Delta H}\right)}_{1}/{\left(\frac{\Delta M}{\Delta H}\right)}_{2}={\left(\frac{\lambda_{2}}{\lambda_{1}}\right)}^{2},$$

where *λ*_1_ and *λ*_2_ are magnetic field penetration depths at temperatures *T*_1_ and *T*_2_, respectively.

**Figure 9:**
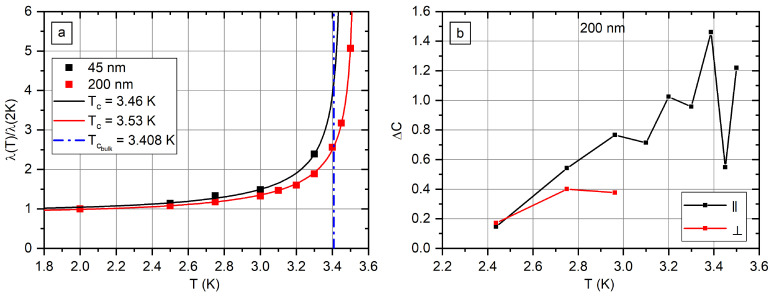
Magnetic field penetration depth as a function of temperature (**a**). The difference in heat capacities Δ*C* of the normal and the superconducting states as a function of temperature (**b**).

Figure 9a shows *λ* as a function of *T* calculated according to (3). Experimental data were fitted with the function:

(4) $$\lambda \left(T\right)=\lambda_{0}{\left(1-{\left(\frac{T}{T_{C}}\right)}^{4}\right)}^{-0.5}$$

The shape of function (4) is in good agreement with the experimental data. Values of *T_C_* are 3.53 K for 200 nm nanowires and 3.46 K for 45 nm nanowires.

Comparing *M*(*H*) curves for s45 and s200, the maximum *M* value for s45 is by one order of magnitude less than the maximum *M* value for s200. However, the magnetic field related to the maximum for the sample s45 is by one order of magnitude more than the magnetic field for s200.

Therefore, the areas under curves s45 and s200 are of the same order of magnitude. Integral of the moment *M* over the field is related to the free energy [55]:

(5) $${\left.\left(F_{n}-F_{s}\right)\right|}_{H=0}=-\int_{0}^{H_{d}}M\left(H\right)dH,$$

where *F_n_* is the free energy of the sample in normal state, *F_s_* is the free energy in superconducting state.

Using the thermodynamic relations:

(6) $$S=-\frac{\partial F}{\partial T}\;$$

(7) $$C=T\frac{\partial S}{\partial T}\;$$

The equation for the heat capacity difference in the normal and superconducting states is as follows:

(8) $$\Delta C={\left.\left(C_{n}-C_{s}\right)\right|}_{H=0}=T\frac{\partial^{2}}{\partial T^{2}}\int_{0}^{H_{d}}M\left(H\right)dH=T\frac{\partial^{2}}{\partial T^{2}}A^{M},$$

where *A^M^* is the designation for the integral of *M* over *H*:

(9) $$A_{i}^{M}=-\int_{0}^{H_{d}}M\left(H,\;T_{i}\right)dH$$

If *A^M^* is measured at three temperatures, *T*_1_, *T*_2_, and *T*_3_, the second order derivative may be calculated according to the equations (see scheme of derivation in Figure S4):

(10) $$T=\frac{T_{1}+2T_{2}+T_{3}}{4}$$

(11) $$\frac{\partial^{2}A}{\partial T^{2}}\left(T\right)=2\frac{\frac{A_{3}^{M}-A_{2}^{M}}{T_{3}-T_{2}}-\frac{A_{2}^{M}-A_{1}^{M}}{T_{2}-T_{1}}}{T_{3}-T_{1}}\;$$

Values of *A^M^* were calculated numerically from the *M*(*H*) curves. The resulting values of ΔC for the sample s200 are shown in Figure 9b. The extrapolation of data to Δ*C* = 0 gives *T* = 2.3 K.

## 4. Conclusions

Superconducting properties of the arrays of parallel In nanowires with the diameters of 45 nm, 200 nm, and 550 nm were measured using SQUID magnetometry. The diameter of 45 nm is several times smaller than the coherence length $\xi_{0_{b}}$, 200 nm is roughly equal to $\xi_{0_{b}}$, and 550 nm is several times greater than $\xi_{0_{b}}$. The method of arrays fabrication eliminates the possibility of bulk indium existence in the samples; XRD data showed that used deposition conditions result in the formation of crystallographically textured nanowires with preferred orientation along <100> axis.

The qualitative difference was observed in *M*(*H*) curves for the nanowires with different diameters. For the arrays of In nanowires with 45 nm and 200 nm diameters, the magnetization curves are completely reversible, increasing field branch is the same as decreasing field branch. For s550 sample, *M*(*H*) curves are irreversible. However, no hysteresis is present in a small field. Presumably, the hysteresis is related to vortex states in the nanowires thick enough. Moreover, characteristic points are observed which may be related to shifts between vortex configurations. The emergence of different configurations is possible if more complicated interaction than repulsion exists between vortices.

Therefore, In, which is a type-I superconductor in bulk state, shows type-II-like behavior if formed in 550 nm nanowires. Nanowires with a diameter less than $\xi_{0_{b}}$ show quasi-type-I behavior: no vortex states are possible because of geometrical limitations. However, the properties of nanowires differ from bulk properties significantly. Critical fields rise with a decrease In nanowire diameter, rise of critical fields up to 20 times compared to the bulk critical field was observed. The increase in critical temperature compared to the bulk values is observed experimentally. The results show wide possibilities for tuning superconducting properties of nanowires by varying diameters and may be useful for applications in nanoelectronics.

Further research in this area could be related to studying the properties of superconducting nanowires in a wider range of diameters to compare the experiment with the theoretical model quantitatively. Another research direction can be aimed to measuring the transport properties of superconducting In nanowires. A change in critical fields by dozens of times should lead to a change in transport properties. Measuring of the transport properties of the structures composed of several In nanowires (e.g., SQUIDs) is also of great interest.

## Figures and Tables

**Table 1:** Parameters of AAO films and polymer track-etched membranes, used as templates.

Sample Name	Type of the Template	Interpore Distance D_int_, nm	Pore Diameter D_p_, nm	Pore Density, μm^−2^	Porosity p, %	Template Thickness, μm	Volume of Pores, 10^−4^ cm^3^
s45	AAO	101 ± 6	45	113	18	40	8.1
s200	AAO	534	200	4.05	12.7	46	6.6
s550	track-etched membrane	—	550	0.34	8	20	1.8

**Figure A1:**
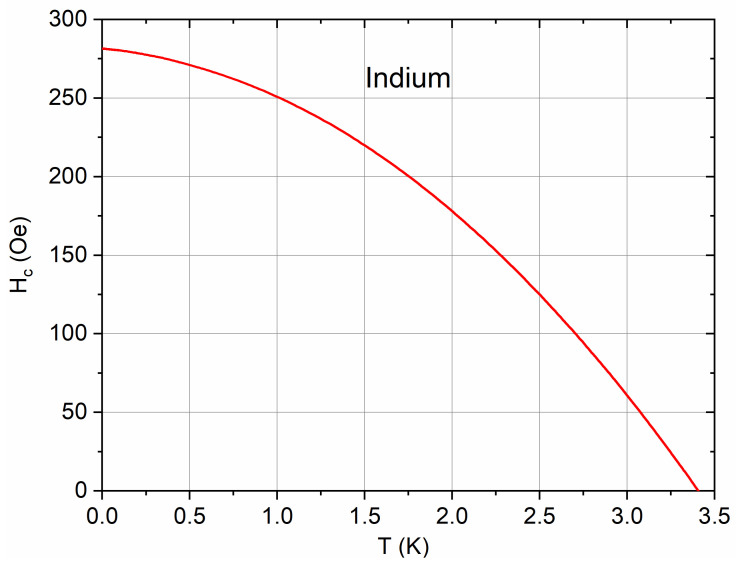
Experimental measurement of the critical field in bulk In as a function of temperature [30].

## Data Availability

The data presented in this study are available on request from the corresponding author.

## Supplementary Materials

The following supporting information can be downloaded at: https://www.mdpi.com/article/10.3390/nano12224095/s1, Figure S1: The magnetic moment *M* as a function of axial magnetic field *H* for the arrays of In nanowires with diameter of 200 nm; Figure S2: The magnetic moment *M* as a function of axial magnetic field *H* for the arrays of In nanowires with diameter of 45 nm; Figure S3: The magnetic moment *M* of the arrays of In nanowires with diameter of 550 nm as a function of magnetic field *H* applied perpendicular to the nanowires; Figure S4: Scheme of Equation (11) derivation.

## Appendix A

**Table A1:** Values of the coherence length and the penetration depth measured in bulk In.

Coherence Length, nm	Penetration Depth, nm	Reference
260		[31]
324		[32]
270		[33]
	70	[34]
	43 ± 2	[35]
200		[36]
	25	[37]
