# Supplementary material for: Size-Dependent Superconducting Properties of In Nanowire Arrays
Source: Nanomaterials (Basel). 2022 Nov 21;12(22):4095. doi: 10.3390/nano12224095 (PMC9695479; doi:10.3390/nano12224095)
Supplement: Supplementary file 1 [file nanomaterials-12-04095-s001.zip › nanomaterials-2010980-supplementary.pdf]

nanomaterials

Article

Supplementary Materials

Size-Dependent Superconducting Properties of in Nanowire Arrays

Alexey A. Noyan [1,2], Yevgeniy A. Ovchenkov [2], Valery V. Ryazanov [3,4], Igor A. Golovchanskiy [1,4], Vasily S. Stolyarov [1,4], Eduard E. Levin [2] and Kirill S. Napolskii [2,4,*]

1 Moscow Institute of Physics and Technology, 141700 Dolgoprudny, Russia;
2 Lomonosov Moscow State University, 119991 Moscow, Russia
3 Institute of Solid State Physics RAS, 142432 Chernogolovka, Russia
4 National University of Science and Technology MISIS, 119049 Moscow, Russia
* Correspondence: kirill@inorg.chem.msu.ru

Citation: Noyan, A.A.; Ovchenkov, Y.A.; Ryazanov, V.V.; Golovchanskiy, I.A.; Stolyarov, V.S.; Levin, E.E.; Napolskii, K.S. Size-Dependent Superconducting Properties of In Nanowire Arrays. *Nanomaterials* **2022**, *12*, 4095. https://doi.org/10.3390/nano12224095

Academic Editors: Gang Xiang and Hongtao Ren

Received: 20 October 2022
Accepted: 14 November 2022
Published: 21 November 2022

Publisher's Note: MDPI stays neutral with regard to jurisdictional claims in published maps and institutional affiliations.

Copyright: © 2022 by the author. Licensee MDPI, Basel, Switzerland. This article is an open access article distributed under the terms and conditions of the Creative Commons Attribution (CC BY) license (https://creativecommons.org/licenses/by/4.0/).

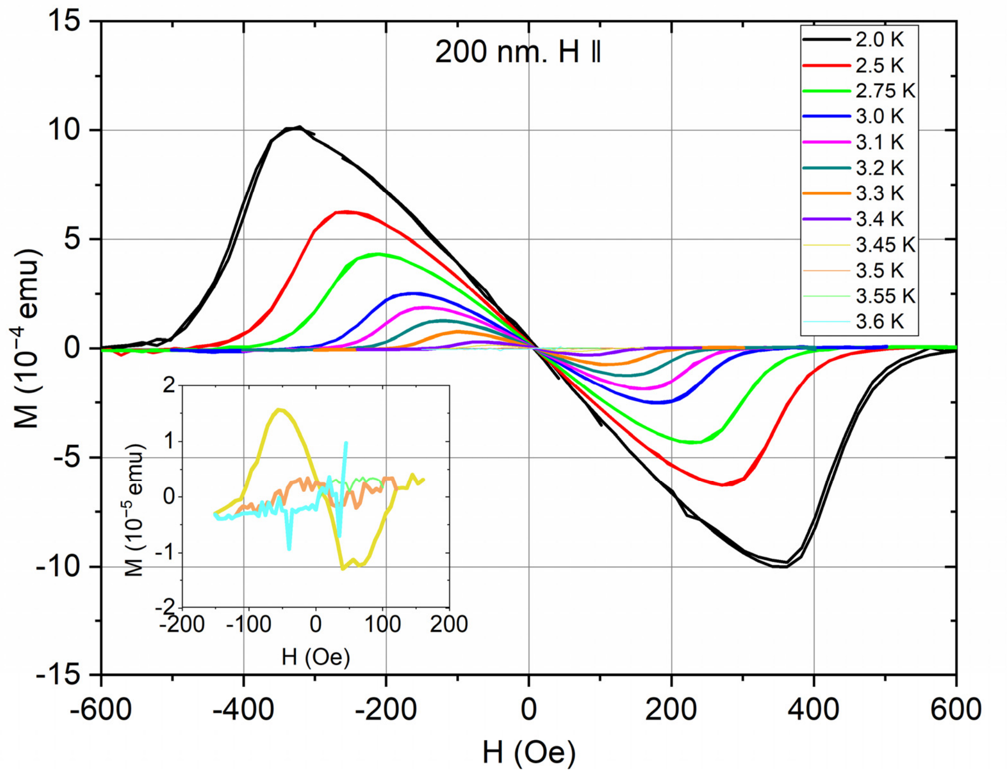

Figure S1. The magnetic moment *M* as a function of axial magnetic field *H* for the arrays of nanowires with diameter of 200 nm.

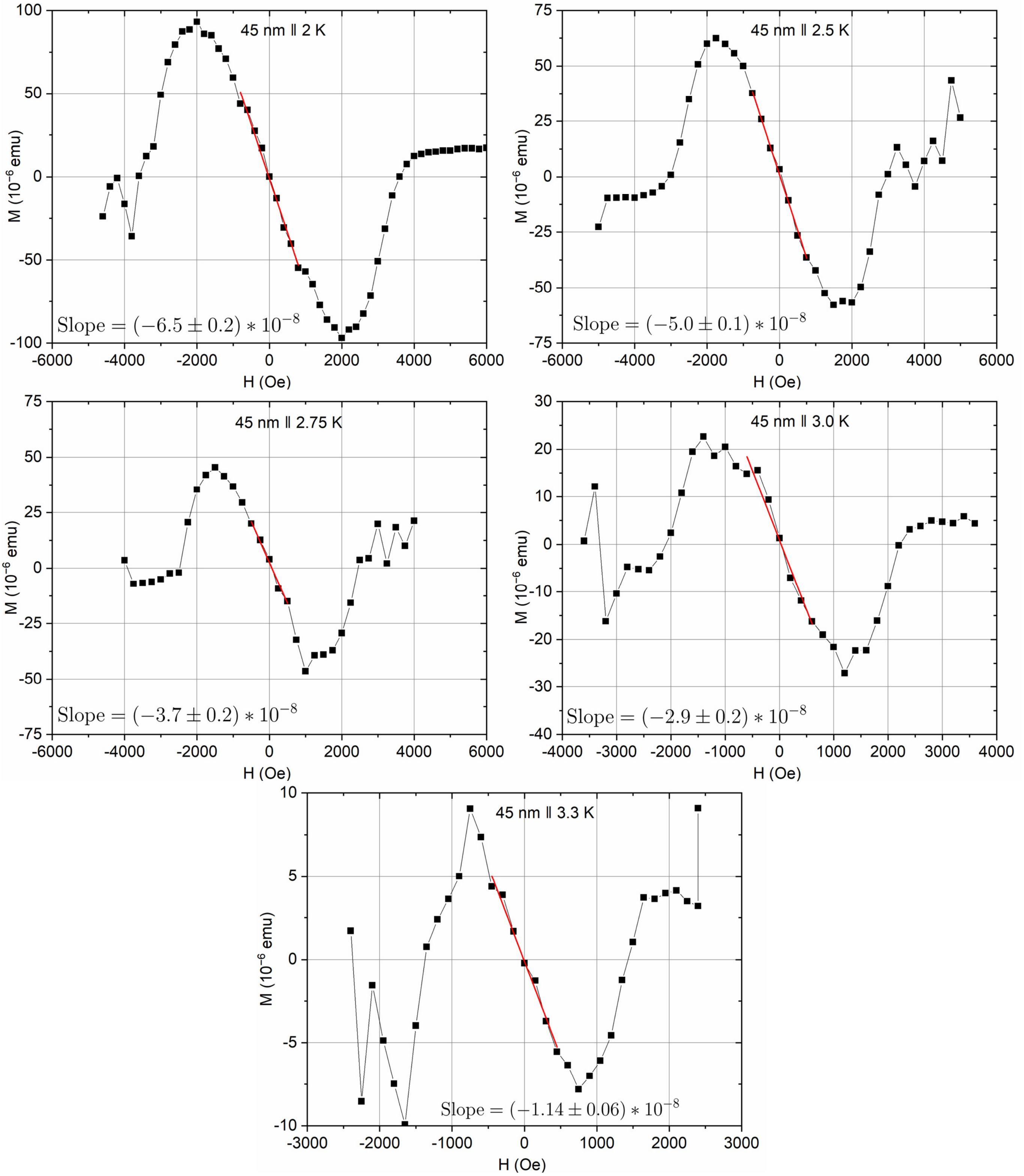

Figure S2. The magnetic moment M as a function of axial magnetic field H for the arrays of nanowires with diameter of 45 nm.

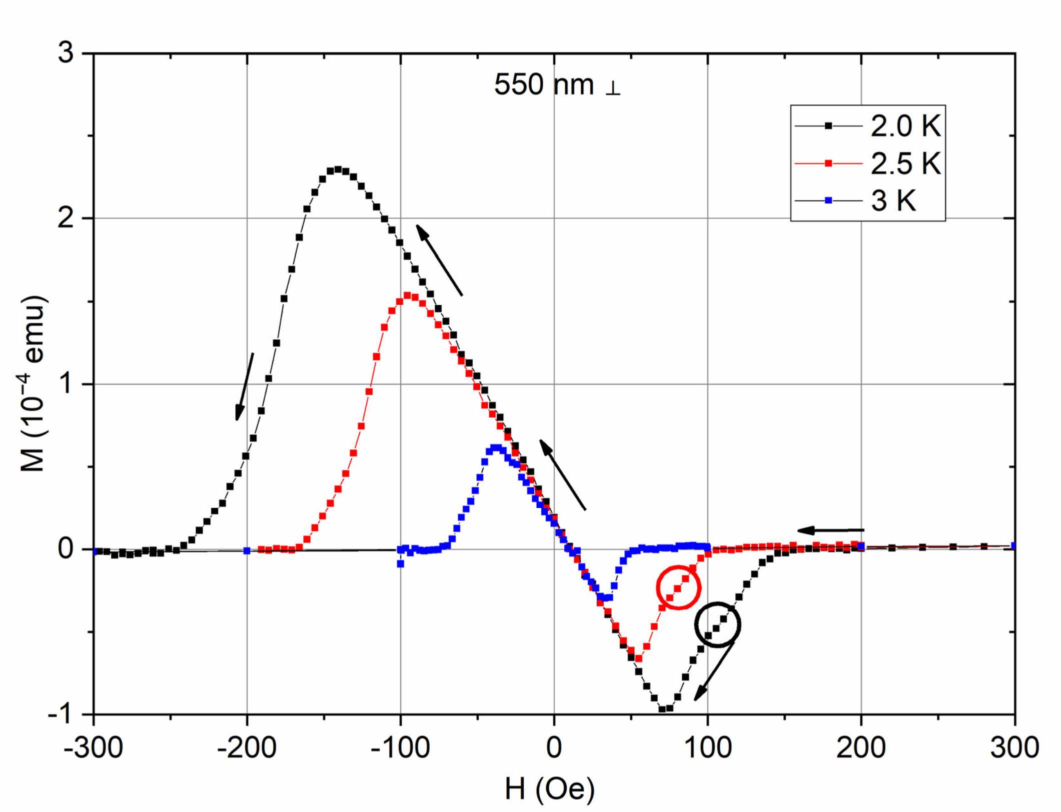

Figure S3. The magnetic moment M of the arrays of nanowires with diameter of 550 nm as a function of magnetic field H applied perpendicular to the nanowires. Arrows show how the field was changing. The features of the curves are marked with circles.

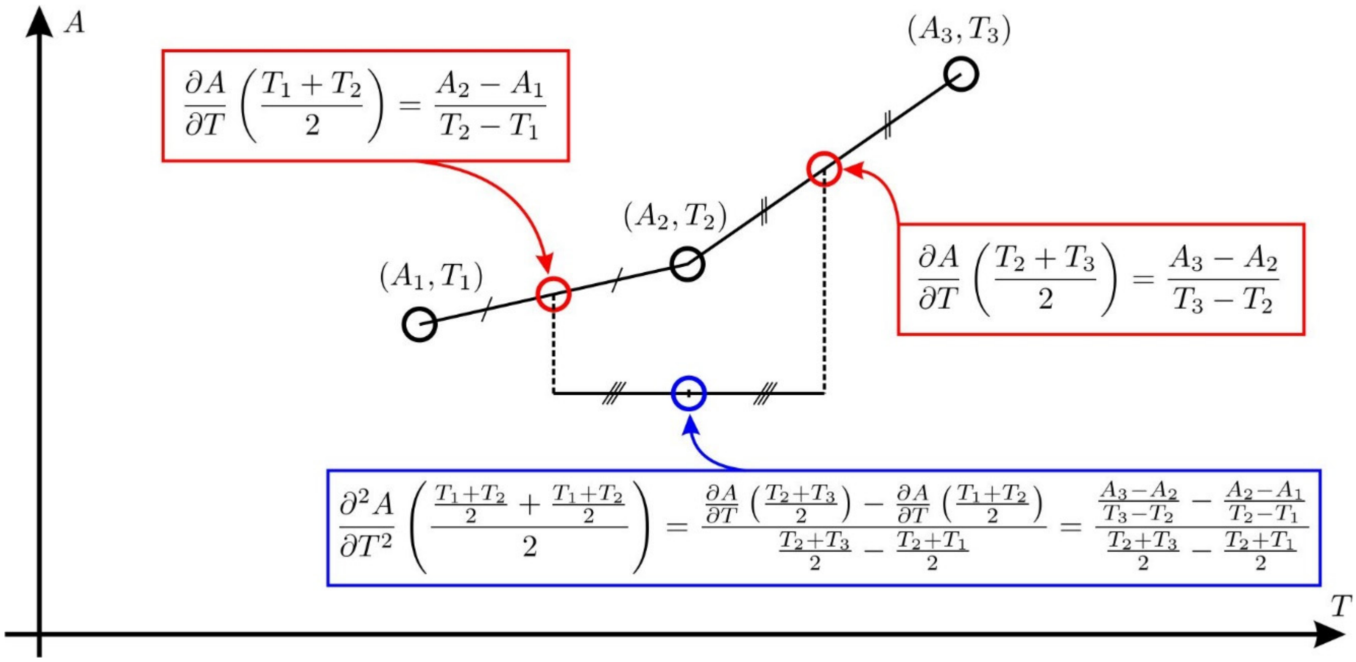

Figure S4. Scheme of equation (11) derivation.
